# Higher serum follistatin levels are associated with increased vascular calcification in haemodialysis patients

**DOI:** 10.3389/fendo.2026.1759582

**Published:** 2026-02-03

**Authors:** Conghui Liu, Aihua Zhang, Zhongxin Li

**Affiliations:** 1 Department of Nephrology, Xuanwu Hospital, Capital Medical University, Beijing, China; 2 Department of Nephrology, Beijing Luhe Hospital, Capital Medical University, Beijing, China

**Keywords:** chronic kidney disease, follistatin, haemodialysis, ROC curve, vascular calcification

## Abstract

**Introduction:**

Follistatin is a secreted protein whose main role is antagonizing the activity of transforming growth factor β (TGF-β) superfamily members. Our study aimed to evaluate the serum follistatin levels and establish their relationship with vascular calcification (VC) in haemodialysis (HD) patients.

**Methods:**

In total, 206 HD patients and 41 healthy individuals, as controls, were included. Serum concentrations of follistatin, along with various clinical and laboratory parameters, were analyzed and compared. VC was assessed using the abdominal aortic calcification (AAC) scores. HD patients were categorized into a low-AAC-score group (AAC score < 4) and a high-AAC-score group (AAC score ≥ 4).

**Results:**

HD patients had higher serum follistatin levels than the controls (1.25 ± 0.53 ng/ml vs. 0.88 ± 0.30 ng/ml, p < 0.001). Compared with the low-AAC-score group, the high-AAC-score group had a higher serum follistatin concentration (1.37 ± 0.57 ng/ml vs. 1.12 ± 0.46 ng/ml, p < 0.001). Multivariate logistic regression revealed that the higher serum follistatin, pulse pressure, serum total cholesterol (TC), serum sodium, and serum corrected calcium were significant independent determinants of high AAC scores of HD patients.

**Conclusion:**

The results herein provide the first clinical evidence of the association between serum follistatin and VC in HD patients. Higher follistatin, pulse pressure, serum corrected calcium levels, and lower serum sodium and TC levels are independent associated with high AAC scores in HD patients.

## 1 Introduction

Vascular calcification (VC) is characterized by the pathological deposition of calcium and phosphate minerals within the extracellular matrix of the vascular wall. Medial calcification is particularly prevalent and clinically significant in patients with chronic kidney disease (CKD). Research demonstrates that vascular calcification is not only highly prevalent but also exhibits a steady progression within the dialysis population (1). In addition, VC significantly elevates the risk of adverse cardiovascular events, stroke, and all-cause mortality in patients with CKD (1–3). However, due to the complex and incompletely understood pathogenesis of VC, evidence supporting many interventions in clinical research remains insufficient or inconsistent, which hinders the development of effective and targeted therapeutic strategies (4). Current evidence suggests that VC in CKD is a complex, actively regulated process involving multiple cell types (5). Therefore, investigating the prevalence and determinants of VC in haemodialysis (HD) patients is essential to developing targeted clinical strategies.

Human follistatin is a secreted protein, predominantly produced by the liver. It is synthesized under both exercise and resting conditions and is regulated by the glucagon-to-insulin ratio (6). Previous studies have demonstrated that the biological functions of follistatin are mediated through its interaction with the transforming growth factor-β (TGF-β) superfamily, including activins, myostatin, and bone morphogenetic protein. Research indicates that follistatin promotes muscle growth in a concentration-dependent manner by antagonizing the negative regulatory effects of myostatin (7). Furthermore, follistatin antagonizes the pathological effects of activin A in the kidneys, including fibrosis, inflammation, and the promotion of cellular aging (8). Another study demonstrated follistatin is capable of recruiting osteoprogenitor cells and enhancing committed osteoblast differentiation and mineralization (9). Based on the role of follistatin in bone metabolism and the bone-vascular cross-talk in patients with CKD, follistatin may be involved in vascular calcification. However, direct experimental evidence is currently lacking. Thus, the purpose of this study was to investigate the level of serum follistatin and the association between follistatin and VC to examine the principal associated factors related to VC in HD patients.

## 2 Materials and methods

### 2.1 Study population

**Figure 1:**
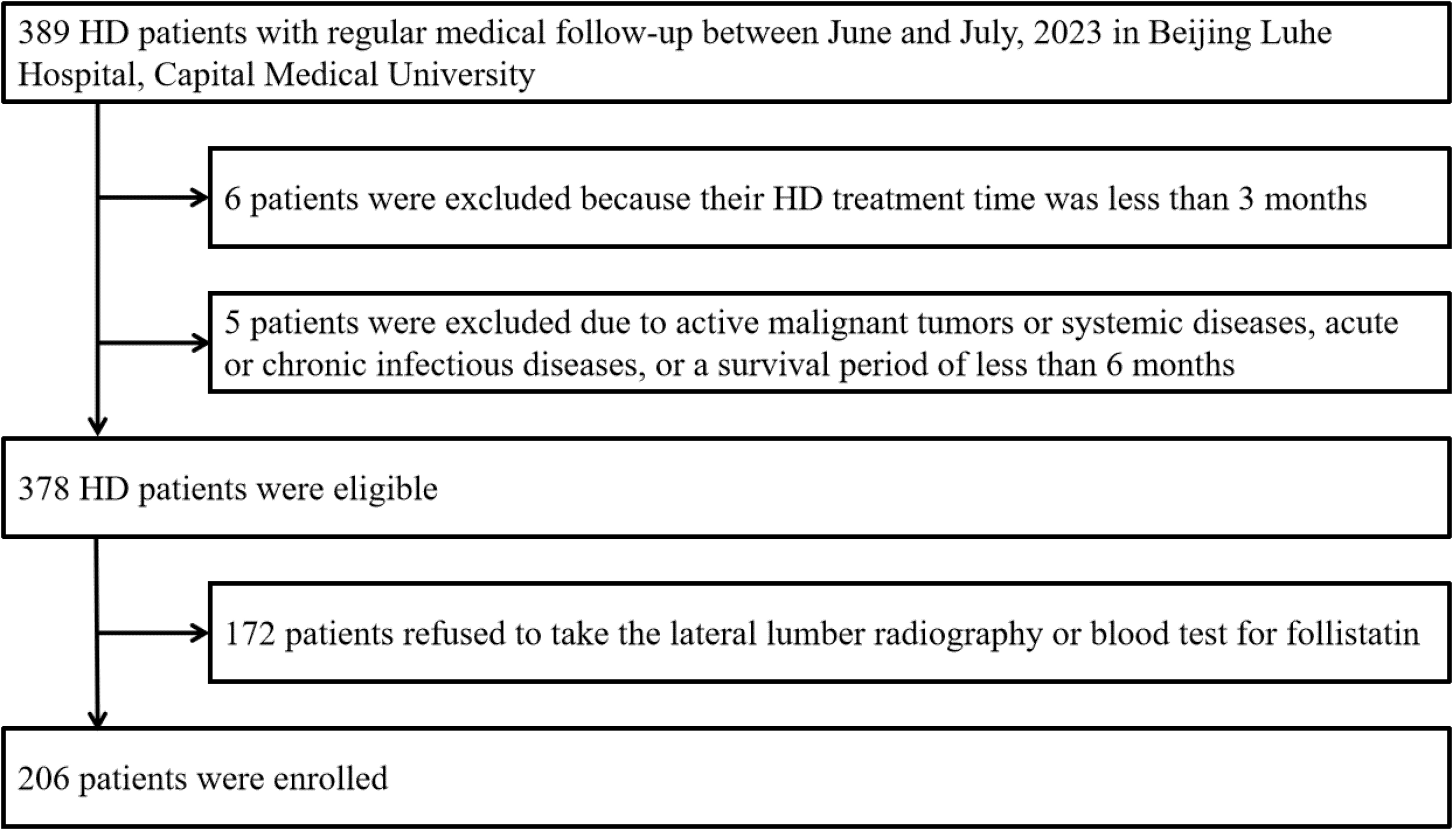
Study flowchart. HD, hemodialysis.

This cross-sectional study screened 389 patients and ultimately enrolled 206 maintenance HD patients at Beijing Luhe Hospital Affiliated with Capital Medical University between June and July 2023. Patients were eligible for inclusion if they (1) were >18 years old, (2) had been on dialysis for at least 3 months, and (3) agreed to participate in this study. The exclusion criteria were as follows: (1) active malignant tumors or systemic diseases, (2) acute or chronic infectious diseases, (3) a survival period of less than 6 months, (4) inability to cooperate with lateral abdominal X-ray due to limb mobility impairment, (5) incomplete clinical data, or unwilling to participate in this study. **Figure 1** shows a flowchart of patient recruitment for the study. We enrolled 41 matched control subjects who underwent routine health checkups at the health medical centre in Beijing Luhe Hospital, Affiliated with Capital Medical University. All participants signed an informed consent form before participating in the study. This clinical study was approved by the Ethics Committee of Beijing Luhe Hospital (2023-LHKY-012-02).

### 2.2 Dialysis procedures

HD patients were treated 3 times per week (3.5–4 h per session) with standard bicarbonate dialysis using a Revaclear 400 dialyzer (with a membrane area of 1.8 m^2^). The vascular access of HD patients is arteriovenous fistula, graft or central venous catheter, with a blood flow rate ranged from 200 to 300 ml/min; the flow rate of the dialysate was 500 ml/min.

### 2.3 Biochemical data collection and measurement of serum follistatin

For all participants, demographics such as age, sex, and the presence of diabetes mellitus were recorded. The following laboratory data were collected at baseline: haemoglobin, leukocytes, platelets, C-reactive protein (CRP), serum albumin, total cholesterol (TC), triglyceride (TG), high-density lipoprotein cholesterol (HDL-C), low-density lipoprotein cholesterol (LDL-C), serum potassium, serum sodium, total carbon dioxide (tCO2), serum creatinine, serum urea, serum uric acid, serum corrected calcium and serum phosphorus.

For HD patients, the following additional information was collected: height, body weight, dialysis vintage, primary kidney disease, history of cardio-cerebrovascular disease (CVD), smoking history, and supplemental biochemical parameters [serum ferritin, intact parathyroid hormone (iPTH), and fractional urea clearance (Kt/V)]. The body mass index (BMI) was calculated as weight/height^2^ (kg/m^2^). Blood pressure was obtained from a single pre-dialysis reading, and pulse pressure was calculated as the difference between systolic and diastolic blood pressure. CVD was recorded if one of the following conditions was present: coronary artery disease, coronary atherosclerotic heart disease, congestive heart failure, transient ischaemic attack, stroke, or peripheral arterial disease (10). The height and body weight were measured.

Blood samples were collected from all participants for measurement of follistatin. For HD patients, blood samples were obtained before haemodialysis treatment. Following centrifugation, the serum was collected and stored at -80 °C until analysis. Serum follistatin concentrations were measured using enzyme-linked immunosorbent assay (ELISA) kits (R&D Systems, Minneapolis, MN, USA) in accordance with the manufacturer’s instructions. The sample was placed in a well of a microplate pre - coated with human follistatin monoclonal antibody and incubated. Subsequently, follistatin antibodies labeled with biotin were added. These antibodies combined with Streptavidin - HRP to form an immune complex. After incubation, the well was washed to remove the unbound enzyme. Then, Chromogen Solution A and B were added.

### 2.4 Assessment of VC

VC was imaged within 2 weeks in enrolled HD patients: abdominal aortic calcification (AAC) by lateral lumbar radiography with Kauppila scoring. Patients were allocated to the high-AAC-score group if they had an AAC score of ≥ 4 (moderate or severe calcification) and to the low-AAC-score group if they had an AAC score of < 4 (no or minor calcification) (11).

### 2.5 Statistical analysis

All analyses were performed using IBM SPSS 26.0 (IBM, USA). Normally distributed quantitative data are presented as the mean ± standard deviation (SD), and nonnormally distributed quantitative data are presented as the median (interquartile range). We performed normality tests on all parameters. For normally distributed data, comparisons between quantitative variables were performed using Student’s t-test or one-way analysis of variance (ANOVA). The Mann–Whitney U test was used to compare two sets of skewed distribution data. The χ^2^ test was used to compare categorical variables. Binary logistic regression analysis was performed to determine the independent factors that affect VC (using forward LR). Variables with *p* values < 0.1 in the univariate logistic regression analysis (serum follistatin, age, with hypertension, pulse pressure, serum TC, serum sodium, serum corrected calcium) were selected. Additionally, variables suspected to be correlated with vascular calcification in clinical practice (even if they had *p* values > 0.1 in the univariate logistic regression analysis, such as dialysis vintage, BMI, serum phosphorus and iPTH) were included in our multivariate logistic regression analysis to identify independent associated factors for a high AAC score in HD patients. A two - tailed *p* value of < 0.05 was considered to indicate a significant difference.

## 3 Results

### 3.1 Characteristics of all study participants

In total, 206 HD patients were recruited according to the inclusion and exclusion criteria. The mean age of those patients was 58.02 ± 11.90 years. The median HD duration was 46.50 (17.00, 83.00) months. The primary renal diseases were diabetes (75 patients, 36.41%), chronic glomerulonephritis (64 patients, 31.07%), hypertensive glomerulosclerosis (36 patients, 17.47%), autosomal dominant polycystic kidney disease (11 patients, 5.34%), and others (20 patients, 9.71%). The vascular access were arteriovenous fistula (195 patients, 94.66%), arteriovenous graft(7 patients, 3.40%) and central venous catheter (4 patients, 1.94%). **Table 1** shows the demographic and clinical characteristics of those patients and 41 healthy controls. HD patients had higher serum follistatin levels than the controls (1.25 ± 0.53 ng/ml vs. 0.88 ± 0.30 ng/ml, *p* < 0.001). Besides, HD patients had lower haemoglobin, platelet, albumin, TC, HDL-C, LDL-C, sodium, tCO2, and corrected calcium levels but higher serum CRP, TG, serum uric acid, serum creatinine, serum urea, serum potassium, and serum phosphorus levels.

**Table 1:** Comparison of parameters between HD patients and normal controls.

Variables	HD (n=206)	Control (n=41)	*P* value
Serum follistatin (ng/ml)	1.25 ± 0.53	0.88 ± 0.30	<0.001
Age (years)	58.02 ± 11.90	55.46 ± 11.76	0.212
Male [n(%)]	126 (61.17%)	22 (53.7%)	0.078
Hypertension [n(%)]	190 (92.23%)	0 (0%)	
Diabetes mellitus [n(%)]	89 (43.20%)	0 (0%)	
CVD history [n(%)]	100 (48.54%)	0 (0%)	
Haemoglobin (g/L)	117.18 ± 12.43	142.80 ± 11.96	<0.001
Leukocytes (*10^9^/L)	5.98 ± 1.89	6.39 ± 1.59	0.192
Platelet (*10^9^/L)	177.03 ± 58.87	247.37 ± 56.11	<0.001
Serum CRP (mg/L)	2.40 (1.00, 5.63)	1.00 (0.49, 2.14)	<0.001
Serum albumin (g/L)	39.47 ± 3.68	45.98 ± 2.22	<0.001
Serum TG (mmol/L)	2.05 ± 1.29	1.35 ± 0.91	<0.001
Serum TC (mmol/L)	3.91 ± 1.04	4.99 ± 0.73	<0.001
Serum HDL-C (mmol/L)	0.95 ± 0.30	1.36 ± 0.29	<0.001
Serum LDL-C (mmol/L)	2.10 ± 0.77	3.14 ± 0.65	<0.001
Serum potassium (mmol/L)	4.78 ± 0.72	4.17 ± 0.35	<0.001
Serum sodium (mmol/L)	137.70 ± 3.22	140.39 ± 1.34	<0.001
Serum tCO2 (mmol/L)	21.75 ± 3.53	25.17 ± 2.51	<0.001
Serum creatinine (μmol/L)	846.28 ± 234.20	72.68 ± 12.86	<0.001
Serum urea (mmol/L)	22.77 ± 6.16	5.02 ± 1.09	<0.001
Serum uric acid (μmol/L)	409.76 ± 86.49	331.00 ± 82.90	<0.001
Serum corrected calcium (mmol/L)	2.13 ± 0.18	2.18 ± 0.09	0.016
Serum phosphorus (mmol/L)	1.71 ± 0.54	1.10 ± 0.14	<0.001

HD, haemodialysis; CVD, cardio-cerebrovascular disease; CRP, c-reactive protein; TG, triglyceride; TC total cholesterol; HDL, high-density lipoprotein cholesterol; LDL-C, low-density lipoprotein cholesterol; tCO2, total carbon dioxide.

### 3.2 Differences in clinical characteristics between HD patients with high serum follistatin and those with low serum follistatin

We divided all dialysis patients into a high-serum-follistatin group (higher than or equal to the mean level) and a low-serum-follistatin group (lower than the mean level) according to the mean follistatin level. As shown in **Table 2**, compared with those in the low-follistatin group, patients in the high-follistatin group had higher AAC scores [9.00 (1.00, 16.00) vs. 2.00 (0.00, 9.00), *p* = 0.019]. A significant difference was observed in the prevalence of moderate or severe vascular calcification (AAC score ≥ 4) between the high and low-follistatin groups (67.82% vs. 42.86%, *p* < 0.001). Besides, patients in the high-follistatin group had longer dialysis vintage, lower BMI, platelets, and serum LDL-C levels. There was no difference in gender, age, diabetes mellitus status, hypertension, CVD history, haemoglobin, leukocyte count, serum CRP, TG, TC, HDL-C, serum albumin, serum sodium, serum potassium, serum tCO2, serum creatinine, serum urea, serum acid, serum corrected calcium, serum phosphate, serum iPTH, spKt/V or serum ferritin between the two groups. Despite non-significantly higher serum albumin levels in the high follistatin group, the prevalence of hypoproteinemia (serum albumin < 40 g/L) was notably higher in this group at 60.92%, compared to 45.38% in the low follistatin group, *p* = 0.027. There was no significant difference in the use of noncalcium phosphate binders, renin-angiotensin-aldosterone system inhibitors, calcitriol, or cinacalcet.

**Table 2:** Comparison of clinical parameters between HD patients with different follistatin levels.

Variables	Low follistatin group (n=119)	High follistatin group (n=87)	*P* value
AAC score	2.00 (0.00, 9.00)	9.00 (1.00, 16.00)	0.019
Age (years)	56.72 ± 11.98	59.80 ± 11.62	0.066
Dialysis vintage (months)	35.00 (16.00, 80.00)	53.00 (19.00, 114.00)	0.044
Male [n(%)]	73 (61.34%)	53 (60.92%)	0.951
Diabetic mellitus [n(%)]	48 (40.34%)	41 (47.13%)	0.331
Hypertension [n(%)]	110 (92.44%)	80 (91.95%)	0.898
CVD history [n(%)]	55 (46.22%)	45 (51.72%)	0.435
Smoking history [n(%)]	44(36.97%)	33 (37.93%)	0.889
BMI (kg/m^2^)	24.38 ± 3.86	23.09 ± 4.25	0.024
Pulse pressure (mmHg)	64.60 ± 17.98	67.20 ± 18.89	0.317
Noncalcium phosphate binders [n(%)]	66 (55.46%)	54 (62.07%)	0.342
RAAS inhibitors [n(%)]	27 (22.69%)	18 (20.69%)	0.732
Calcitriol [n(%)]	72 (60.50%)	51 (58.62%)	0.785
Cinacalcet [n(%)]	38 (31.93%)	24 (27.59%)	0.502
Haemoglobin (g/L)	117.65 ± 12.39	116.54 ± 12.52	0.529
Leukocytes (*10^9^/L)	6.03 ± 2.03	5.92 ± 1.68	0.679
Platelets (*10^9^/L)	185.76 ± 58.76	165.10 ± 57.23	0.013
Serum CRP (mg/L)	2.50 (10.98, 6.84)	2.20 (1.12, 4.51)	0.687
Serum TG (mmol/L)	1.84 (1.12, 2.50)	1.67 (1.15, 2.64)	0.661
Serum TC (mmol/L)	4.01 ± 1.00	3.78 ± 1.07	0.119
Serum HDL-C (mmol/L)	0.94 ± 0.28	0.96 ± 0.32	0.648
Serum LDL-C (mmol/L)	2.20 ± 0.87	1.95 ± 0.79	0.022
Serum albumin (g/L)	39.85 ± 3.86	38.95 ± 3.38	0.082
Serum potassium (mmol/L)	4.72 ± 0.70	4.86 ± 0.73	0.163
Serum sodium (mmol/L)	137.93 ± 2.96	137.38 ± 3.53	0.236
Serum tCO2 (mmol/L)	21.97 ± 3.36	21.45 ± 3.75	0.302
Pre-hemodialysis serum creatinine (μmol/L)	855.08 ± 246.60	834.24 ± 216.92	0.521
Pre-hemodialysis serum urea (mmol/L)	22.33 ± 6.27	23.38 ± 65.99	0.229
Serum uric acid (μmol/L)	413.08 ± 83.51	405.23 ± 90.70	0.521
Serum corrected calcium (mmol/L)	2.15 ± 0.18	2.11 ± 0.18	0.178
Pre-hemodialysis serum phosphorus (mmol/L)	1.71 ± 0.53	1.72 ± 0.55	0.894
Serum iPTH (pg/ml)	227.10 (125.00, 333.20)	198.30 (104.10, 290.90)	0.318
Serum ferritin (ng/ml)	340.90 (150.55, 492.19)	330.50 (185.53, 525.50)	0.942
spKt/V (single pool, per time)	1.42 ± 0.27	1.49 ± 0.43	0.205

HD, haemodialysis; AAC, abdominal aortic calcification; CVD, cardio-cerebrovascular disease;; BMI, body mass index; RAAS, renin-angiotensin-aldosterone system; CRP, C-reactive protein; TG, triglyceride; TC, total cholesterol; HDL, high-density lipoprotein cholesterol; LDL-C, low-density lipoprotein cholesterol; tCO2, total carbon dioxide; iPTH, intact parathyroid hormone.

### 3.3 Comparison of follistatin levels and other parameters between patients with high AAC scores and those with low AAC scores

The median calcification score of 206 HD patients was 4.00 (interquartile range: 0.00 - 12.00). Of these, 110 patients exhibited moderate to severe vascular calcification, as defined by an AAC score ≥ 4 points. Compared with the low-AAC-score group, the high-AAC-score group had a higher serum follistatin concentration (1.37 ± 0.57 ng/ml vs. 1.12 ± 0.46 ng/ml, *p* < 0.001). HD patients with high AAC scores had lower serum sodium levels than patients with low AAC scores (*p* < 0.05). However, age, CVD history, pulse pressure, and serum corrected calcium were significantly greater in HD patients with a high AAC score than in those with a low AAC score (*p* < 0.05). In addition, there were no differences in dialysis duration, gender, BMI, haemoglobin, leukocyte count, platelets, serum CRP, TG, TC, HDL-C, LDL-C, serum albumin, serum potassium, serum creatinine, serum urea, serum phosphorus, serum iPTH, or serum ferritin between the high-AAC-score group and the low-AAC-score group. In terms of medication history, 74.55% of patients in the high-AAC-score group were taking noncalcium phosphate binders, which was significantly greater than the percentage in the low-AAC-score group (39.58%). There was no difference in the use of renin-angiotensin-aldosterone system inhibitors, calcitriol, or cinacalcet (**Table 3**).

**Table 3:** Comparison of follistatin levels and other parameters between HD patients with high AAC scores and those with low AAC scores.

Variables	Low AAC Score (n=96)	High AAC Score (n=110)	*P* value
Serum follistatin (ng/ml)	1.12 ± 0.46	1.37 ± 0.57	<0.001
Age (years)	55.91 ± 12.69	59.87 ± 10.89	0.017
Dialysis vintage (months)	36.00 (13.25, 73.75)	54.50 (19.75, 97.50)	0.050
Male [n(%)]	59 (61.46%)	67 (60.91%)	0.936
Diabetes mellitus [n(%)]	37 (38.54%)	52 (47.27%)	0.207
Hypertension [n(%)]	85 (88.54%)	105 (95.45%)	0.064
CVD history [n(%)]	36 (37.50%)	64 (58.20%)	0.003
Smoking history [n(%)]	38(39.58%)	39 (35.45%)	0.541
BMI (kg/m^2^)	23.81 ± 4.09	23.86 ± 4.06	0.923
Pulse pressure (mmHg)	62.54 ± 15.90	68.45 ± 19.95	0.019
Noncalcium phosphate binders [n(%)]	38 (39.58%)	82 (74.55%)	<0.001
RAAS inhibitors [n(%)]	20 (20.83%)	25 (22.73%)	0.743
Calcitriol [n(%)]	61 (63.54%)	62 (56.36%)	0.295
Cinacalcet [n(%)]	29 (30.21%)	33 (30.00%)	0.974
Haemoglobin (g/L)	117.88 ± 13.53	116.57 ± 11.40	0.454
Leukocytes (*10^9^/L)	6.02 ± 1.79	5.95 ± 1.98	0.787
Platelets (*10^9^/L)	183.18 ± 59.33	171.70 ± 58.21	0.162
Serum CRP (mg/L)	2.45 (1.12, 6.15)	2.40 (0.97, 5.18)	0.555
Serum TG (mmol/L)	1.87 (1.15, 2.58)	1.67 (1.12, 2.51)	0.383
Serum TC (mmol/L)	4.06 ± 0.98	3.79 ± 1.07	0.059
Serum HDL-C (mmol/L)	0.96 ± 0.33	0.94 ± 0.27	0.631
Serum LDL-C (mmol/L)	2.19 ± 0.71	2.02 ± 0.82	0.120
Serum albumin (g/L)	39.89 ± 3.76	39.09 ± 3.59	0.119
Serum potassium (mmol/L)	4.75 ± 0.74	4.81 ± 0.70	0.540
Serum sodium (mmol/L)	138.25 ± 3.25	137.22 ± 3.13	0.021
Serum tCO2 (mmol/L)	21.47 ± 3.54	22.00 ± 3.52	0.279
Serum creatinine (μmol/L)	854.73 ± 235.41	838.91 ± 233.97	0.630
Serum urea (mmol/L)	22.34 ± 6.40	23.15 ± 5.95	0.350
Serum uric acid (μmol/L)	413.61 ± 96.39	406.40 ± 77.11	0.552
Serum corrected calcium (mmol/L)	2.11 ± 0.19	2.16 ± 0.18	0.040
Serum phosphorus (mmol/L)	1.70 ± 0.56	1.72 ± 0.52	0.850
Serum iPTH (pg/ml)	198.70 (103.20, 310.20)	224.70 (114.10, 323.05)	0.568
spKt/V (single pool, per time)	1.45 ± 0.26	1.46 ± 0.40	0.727
Serum ferritin (ng/ml)	327.20 (155.90, 490.08)	337.60 (182.25, 518.24)	0.600

HD, haemodialysis; AAC, abdominal aortic calcification; CVD, cardio-cerebrovascular disease; BMI, body mass index; RAAS, renin–angiotensin–aldosterone system; CRP, C-reactive protein; TG, triglyceride; TC, total cholesterol; HDL, high-density lipoprotein cholesterol; LDL-C, low-density lipoprotein cholesterol; tCO2, total carbon dioxide; iPTH, intact parathyroid hormone.

### 3.4 Independent determining factors for vascular calcification according to multiple logistic regression analysis in HD patients

To eliminate the impact of confounding factors on vascular calcification, a multivariate logistic regression model was used to identify the independent associated factors for vascular calcification in HD patients. Variables with *p* values < 0.1 in the univariate logistic regression analysis were selected. Additionally, variables suspected to be correlated with vascular calcification in clinical practice (even if they had *p* values > 0.1 in the univariate logistic regression analysis, such as dialysis vintage, BMI, serum phosphorus and iPTH) were included in our multivariate logistic regression analysis to identify independent associated factors for a higher AAC score in HD patients. In the analysis, higher serum follistatin, higher pulse pressure, lower serum TC, lower serum sodium, and higher serum corrected calcium were independently associated with vascular calcification in HD patients (**Table 4**).

**Table 4:** Independent determining factors for a high AAC score according to the multiple logistic regression analysis in HD patients.

Variable	Univariate logistic regression		Multivariate logistic regression	
	B (95% CI)	*P* value	B (95% CI)	*P* value
Serum follistatin (ng/ml)	2.798 (1.511 to 5.181)	0.001	2.974 (1.560 to 5.668)	0.001
Age (years)	1.029 (1.005 to 1.054)	0.018	Unentered	
Dialysis vintage (months)	1.004 (0.999 to 1.008)	0.114	Unentered	
With diabetes mellitus (no = 0, yes = 1)	1.430 (0.820 to 2.493)	0.208		
With hypertension (no = 0, yes = 1)	2.718 (0.909 to 8.125)	0.074	Unentered	
BMI (kg/m^2^)	1.003 (0.938 to 1.073)	0.923	Unentered	
Pulse pressure (mmHg)	1.019 (1.003 to 1.035)	0.023	1.019 (1.002 to 1.037)	0.033
Serum TC (mmol/L)	0.771 (0.587 to 1.012)	0.061	0.732 (0.548 to 0.979)	0.035
Serum sodium (mmol/L)	0.902 (0.825 to 0.986)	0.023	0.903 (0.822 to 0.992)	0.034
Serum corrected calcium (mmol/L)	5.004 (1.060 to 23.620)	0.042	8.824 (1.642 to 47.411)	0.011
Serum phosphorus (mmol/L)	1.050 (0.631 to 1.748)	0.850	Unentered	
Serum iPTH (pg/ml)	1.001(0.999 to 1.002)	0.353	Unentered	

AAC, abdominal aortic calcification; BMI, body mass index; HD, haemodialysis; TC total cholesterol; iPTH, intact parathyroid hormone; CI, confidence interval.

### 3.5 The predictive ability of follistatin for vascular calcification in HD patients

**Figure 2:**
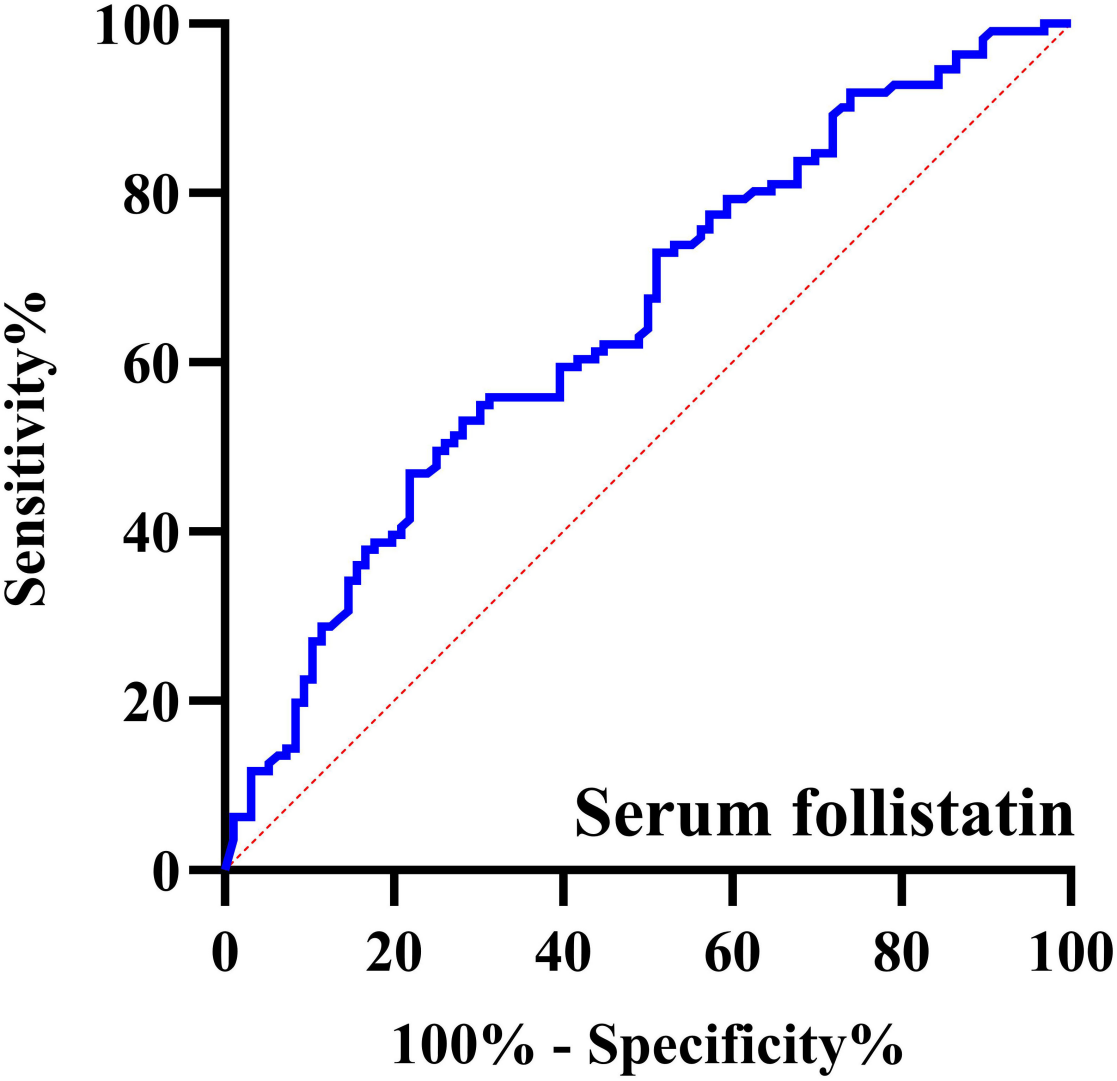
ROC curve of the serum follistatin concentration for predicting vascular calcification in hemodialysis patients.

The ROC curve analyzing serum follistatin concentration for predicting vascular calcification in HD patients yielded an AUC of 0.655 (95% CI: 0.580-0.729; *p* < 0.001). At the optimal cut-off value of 1.26 ng/mL, the sensitivity was 53.6% and the specificity was 71.9% (**Figure 2**).

## 4 Discussion

In our study, 53.40% of the HD patients exhibited moderate to severe AAC, as determined using X-ray plain films. Our findings represent the first demonstration that serum follistatin levels are markedly elevated in HD patients compared to healthy individuals. Furthermore, HD patients with high AAC scores displayed significantly increased serum follistatin concentrations relative to those with lower AAC scores. Multivariate logistic regression analysis further revealed that elevated serum follistatin was an independent predictor of VC in HD patients.

Unlike in healthy adults, urinary follistatin levels were significantly elevated in patients with acute kidney injury, showing a positive correlation with disease severity. Notably, the highest follistatin levels were observed in those requiring renal replacement therapy (12). This pattern suggests that circulating follistatin is not primarily cleared by the kidneys. However, the association between follistatin and renal function has shown inconsistent findings across previous studies. Studies in animal models have demonstrated that follistatin exerts protective effects in various models of kidney injury, including diabetic nephropathy, acute kidney injury, and CKD (8, 13, 14). A prospective cohort study involving 4733 individuals demonstrated that an increase in serum follistatin levels occurs more than 20 years before CKD manifestation, and confirmed that baseline follistatin is independently associated with the incidence of CKD (15). However, in another study involving 328 patients with CKD and 32 participants without CKD, no significant difference was observed in follistatin levels between patients with stage 5 CKD and those with stages 1–4 CKD or non-CKD individuals (16). In our study, HD patients have greater follistatin concentration than controls. Previous studies have demonstrated that circulating follistatin levels are elevated under conditions including acute and chronic energy deficiency (17), type 2 diabetes (18), polycystic ovary syndrome (19), cancer (20), and chronic inflammation (21), indicating a potential association between increased follistatin concentrations and metabolic dysregulation. So, we speculate that the elevated follistatin levels observed in HD patients may be associated with those conditions.

A further finding was that patients in the high-follistatin group had a longer dialysis vintage and lower BMI, platelet counts, and LDL-C. Although the difference in serum albumin levels between the two groups did not reach statistical significance, the prevalence of hypoalbuminemia was markedly higher in the high-follistatin group (60.92% vs. 45.38%, p = 0.027). These findings imply that elevated follistatin levels may be associated with poorer nutritional status in HD patients. This is consistent with another study in a CKD cohort, which showed that CKD stage 5 patients with high follistatin levels presented with lower serum albumin, serum creatinine, and grip strength, but elevated interleukin-6 and CRP levels, as well as a greater prevalence of protein-energy wasting (16). The association between serum follistatin and platelet count has not been documented in clinical studies. However, follistatin indirectly negatively regulates platelet production by inhibiting the bone morphogenetic protein 4 (a member of the TGF-β family) signaling pathway *in vitro (*22).

VC is common among the dialysis population. A 4-year cohort study of 1,489 dialysis patients revealed a marked increase in VC prevalence, from 71% at baseline to 90.7% (1). The incidence and progression of vascular calcification vary according to anatomical location, with coronary arteries showing a higher prevalence of calcification, while the AAC exhibits the most pronounced disease progression (1). The prevalence of AAC among HD patients has been reported to range from 49.6% to 88.6% across different studies (1, 23). The traditional risk factors for VC in dialysis patients include advanced age, longer dialysis vintage, diabetes mellitus, mineral and bone disorder (e.g., hyperphosphatemia, hypercalcaemia or increased calcium load, high iPTH levels), and inflammatory states. We also found that elevated levels of serum corrected calcium are significantly associated with an increased risk of VC. In addition, elevated pulse pressure, the difference between systolic and diastolic blood pressure, is an indicator of arterial stiffness and is independently associated with VC. Evidence confirms a positive correlation between pulse pressure and AAC (24), while greater carotid calcification volume predicts a steeper rise in pulse pressure over time (25). The relationship between pulse pressure and VC may be bidirectional. On one hand, increased pulse pressure may reflect more extensive arterial calcification and reduced vascular compliance. On the other hand, chronically elevated pulse pressure may contribute to the initiation and progression of vascular calcification. Both serum sodium and TC levels exhibit a U-shaped relationship with the prognosis of HD patients (26, 27); the optimal range of these for reducing VC and all-cause mortality remains unclear. In our study, the association between serum sodium, TC levels, and VC may be attributable to underlying malnutrition.

The most important finding of our study was that increased serum follistatin concentrations were independently associated with increased VC in HD patients. A previous study demonstrated that activin A treatment strongly inhibited mineralization in osteoblast cultures, whereas the activin antagonist follistatin increased mineralization, but follistatin treatment did not alter gene expression in vascular smooth muscle cells (28). Overexpression of follistatin in growing mice leads to decreased quality of skeleton, increasing susceptibility to bone fractures (29). A clinical trial found that follistatin levels were strongly negatively associated with bone mineral density in patients with CKD (16). Therefore, follistatin may be involved in bone metabolic disorders; however, the exact role of follistatin in VC in HD patients must be further explored through *in vitro* and *in vivo* experiments. Given the established correlations between follistatin and markers of malnutrition and inflammation, we hypothesize that the association between elevated follistatin levels and VC may be mediated by malnutrition and inflammation.

The present study has certain limitations. Firstly, the number of serum samples analyzed was relatively small. Secondly, due to the cross-sectional design of the study, it was not possible to establish a causal relationship between follistatin and VC in HD patients.

## 5 Conclusion

Our results are the first to provide clinical evidence of the association between serum follistatin and VC in HD patients. Higher follistatin, pulse pressure, serum corrected calcium levels, and lower serum sodium and lower TC levels are independent associated factors for VC in HD patients. These findings indicate that follistatin may be involved in the pathophysiology of VC and warrant further basic and clinical research.

## Data Availability

The raw data supporting the conclusions of this article will be made available by the authors, without undue reservation.
